# Glucocorticoids Induce Stress Oncoproteins Associated with Therapy-Resistance in African American and European American Prostate Cancer Cells

**DOI:** 10.1038/s41598-018-33150-2

**Published:** 2018-10-10

**Authors:** Leanne Woods-Burnham, Christina K. Cajigas-Du Ross, Arthur Love, Anamika Basu, Evelyn S. Sanchez-Hernandez, Shannalee R. Martinez, Greisha L. Ortiz-Hernández, Laura Stiel, Alfonso M. Durán, Colwick Wilson, Susanne Montgomery, Sourav Roy, Carlos A. Casiano

**Affiliations:** 1 Center for Health Disparities and Molecular Medicine and Department of Basic Sciences, Loma Linda University School of Medicine, Loma Linda, CA USA; 2 Loma Linda University School of Behavioral Health, Loma Linda, CA USA; 3 Oakwood University, Huntsville, AL USA; 4 Department of Entomology and Institute for Integrative Genome Biology, University of California Riverside, Riverside, CA USA; 5 Department of Medicine, Loma Linda University School of Medicine, Loma Linda, CA USA

## Abstract

Glucocorticoid receptor (GR) is emerging as a key driver of prostate cancer (PCa) progression and therapy resistance in the absence of androgen receptor (AR) signaling. Acting as a bypass mechanism, GR activates AR-regulated genes, although GR-target genes contributing to PCa therapy resistance remain to be identified. Emerging evidence also shows that African American (AA) men, who disproportionately develop aggressive PCa, have hypersensitive GR signaling linked to cumulative stressful life events. Using racially diverse PCa cell lines (MDA-PCa-2b, 22Rv1, PC3, and DU145) we examined the effects of glucocorticoids on the expression of two stress oncoproteins associated with PCa therapy resistance, Clusterin (CLU) and Lens Epithelium-Derived Growth Factor p75 (LEDGF/p75). We observed that glucocorticoids upregulated LEDGF/p75 and CLU in PCa cells. Blockade of GR activation abolished this upregulation. We also detected increased GR transcript expression in AA PCa tissues, compared to European American (EA) tissues, using Oncomine microarray datasets. These results demonstrate that glucocorticoids upregulate the therapy resistance-associated oncoproteins LEDGF/p75 and CLU, and suggest that this effect may be enhanced in AA PCa. This study provides an initial framework for understanding the contribution of glucocorticoid signaling to PCa health disparities.

## Introduction

For decades, androgen deprivation therapy (ADT) has been a mainstay of treatment for advanced prostate cancer (PCa)^1–4^. The mechanism of action of ADT involves the lowering of serum testosterone or competitively blocking the binding of androgens to androgen receptor (AR). However, this therapy is not curative, as several studies have conclusively demonstrated that prostate tumors develop ADT-resistance^2,3^. Glucocorticoid receptor (GR) signaling has recently been shown to drive ADT-resistance via its ability to bypass the AR pathway blockade and directly restore activation of AR-target genes in addition to activating an independent transcriptome that also drives therapy resistance^2,5–10^.

A pressing implication is that glucocorticoid therapy presently administered to PCa patients as a standard of care could be detrimental under certain clinical conditions^2,5,10–12^. For example, there is evidence that glucocorticoids promote PCa progression in patients whose tumors express GR, and that men who receive glucocorticoids concomitantly with the second-line ADT drug enzalutamide have worse overall survival^5,7^. However, a clinical dilemma exists as glucocorticoids confer many palliative benefits to patients who often suffer from debilitating side effects of their treatment^13^.

Similarly, the importance of glucocorticoid co-therapy also extends to taxane-based chemotherapeutic regimens for patients with metastatic castration-resistant PCa (mCRPC). The taxane drugs docetaxel (DTX) and cabazitaxel (CTX) can extend patient survival, however, they are also not curative because patients eventually develop resistance to these drugs^14,15^. Glucocorticoids are commonly co-administered with taxanes to mitigate side effects of chemotherapy such as nausea, vomiting, and inflammatory reactions. Of concern, however, is the recent evidence pointing to the possible contribution of GR signaling to the acquisition of taxane resistance in breast and prostate cancers^16,17^.

While the ability of GR to activate AR-target genes in the context of mCRPC has been demonstrated^2,5–12^, there is a need to identify specific genes driven by GR signaling that have been previously linked to taxane chemotherapy. This is critical to our understanding of mechanisms by which GR may induce taxane resistance, and the identification of potential therapeutic targets. We hypothesized that stress oncoproteins that are upregulated in the context of standard PCa treatments and that promote therapy resistance may be upregulated by GR signaling. As a first step in evaluating this hypothesis we focused on the contribution of GR signaling to the expression of the stress oncoproteins Clusterin (CLU) and Lens Epithelium-Derived Growth Factor p75 (LEDGF/p75), previously shown to be upregulated in response to standard PCa therapies, including taxane therapy^18–28^. CLU is an AR-regulated, anti-apoptotic protein that is upregulated in PCa, particularly following ADT, as well as several other cancers^19,21,23,24,29–35^. CLU has two isoforms that result from two transcriptional start sites; nuclear CLU is pro-apoptotic and sequestered in the nucleus whereas secreted CLU (sCLU) is ultimately secreted following post-translational modifications and cleavage into two distinct alpha and beta peptides held together by disulfide bonds^19,20,36^. Before cleavage, sCLU exists in the cytoplasm as a pre-secreted form (psCLU) and both forms contribute to DTX resistance^20,22^. The role of CLU in mCRPC resistance to DTX is also well defined^18,20,22,25,37^.

Similar to CLU, LEDGF/p75 also promotes taxane resistance in PCa cells, albeit by a different mechanism. Our group and others have demonstrated that LEDGF/p75 is a stress response transcription co-activator upregulated in PCa as well as other cancers that promotes cellular survival in the presence of chemotherapeutic drugs and radiation^27,28,38–45^. While CLU inhibits drug-induced apoptosis by preventing mitochondrial membrane permeabilization^20,22,23^, LEDGF/p75, acting as a stress transcription co-activator, transactivates stress response and anti-oxidant genes, and promotes resistance to oxidative stress-induced necrosis and DTX-induced caspase-independent lysosomal cell death^27,28,38,39^. In a recent study, we showed that depletion of LEDGF/p75 in DTX-resistant mCRPC cells partially resensitized the cells to DTX treatment^28^. Further, other groups have shown that downregulation of LEDGF/p75 reduced cancer cell proliferation, migration, tumorigenicity, and sensitized cancer cells to anti-tumor drugs^46–49^. In addition to its roles in cancer, LEDGF/p75 also functions as a key cellular co-factor of HIV-1 integration, and as an autoantigen (called DFS70) targeted by autoantibodies in subsets of healthy individuals and patients with miscellaneous inflammatory conditions^44^.

The implications of glucocorticoid-activated GR signaling upregulating oncoproteins associated with tumor progression and therapy resistance are far-reaching and have the potential to impact PCa patients who may have elevated levels of endogenous cortisol or a propensity for hypersensitive GR signaling. For example, individuals exposed to chronic stressful life events tend to have increased cortisol levels, which have been positively associated with increased exposure to psychosocial stressors^50–52^. This could be problematic for African American (AA) men, who bear a disproportionate burden of incidence and mortality of PCa, compared to European American (EA) men^53–62^, and have been shown to have increased cortisol production directly linked to cumulative stressful life events^51^. In addition, there is evidence of dysregulated GR signaling due to hypersensitive GRs in AA men^52,63^. Recent studies demonstrated that the potent glucocorticoid treatment, which is included in PCa therapy regimens^13,64,65^, induced dynamic changes in CpG methylation as well as transcription of neighboring genes within an AA cohort, and disease enrichment analysis of Dex-induced genes revealed associations with aging-related diseases including cancers^52^.

It is well established that AA men are diagnosed with a more aggressive PCa phenotype attributable to the interplay between a number of factors including socioeconomic status, access to healthcare, diet and lifestyle, and biological contributors^53–61,66–83^. However, it has yet to be explored whether the endogenously elevated glucocorticoid signaling in AA men that has been linked to cumulative stressful life events plays a role in the aggressive PCa phenotype and mortality disparities observed in this population. In this overall context, we examined the contribution of GR signaling to upregulation of LEDGF/p75 and CLU in a racially diverse panel of PCa cell lines, and explored the transcript expression of GR in racially diverse PCa tissues.

## Results

### Activated AR signaling upregulates LEDGF/p75 and CLU in 22Rv1 cells

The GR bypass hypothesis posits that GR takes over the regulation of AR-target genes after treatment of advanced PCa with primary and secondary ADT^2^. Although CLU is known to be androgen regulated^19,21,23^, there are no studies determining that LEDGF/p75 expression is also stimulated by androgen exposure in PCa cells. To explore this, we examined the protein expression levels of LEDGF/p75 and CLU in androgen responsive 22Rv1 PCa cells following exposure to dihydrotestosterone (DHT) and the secondary ADT drug enzalutamide (Enz). We treated cells with either 1 nM DHT, 10 nM DHT, or 1 μM Enz for 24 hours, and collected total lysates of both treated and untreated cells to perform immunoblotting analyses using antibodies specific for either LEDGF/p75 or CLU. We observed increased protein levels of both LEDGF/p75 and CLU in cells treated with either 1 nM or 10 nM DHT, which was attenuated by exposure to 1 μM Enz (Supplementary Fig. 1). These results were consistent with the previously reported upregulation of CLU in PCa cells by androgens, and also provided evidence for androgen induction of LEDGF/p75 in PCa cells.

### Activated GR signaling upregulates LEDGF/p75 and CLU protein expression in PCa cells

**Figure 1:**
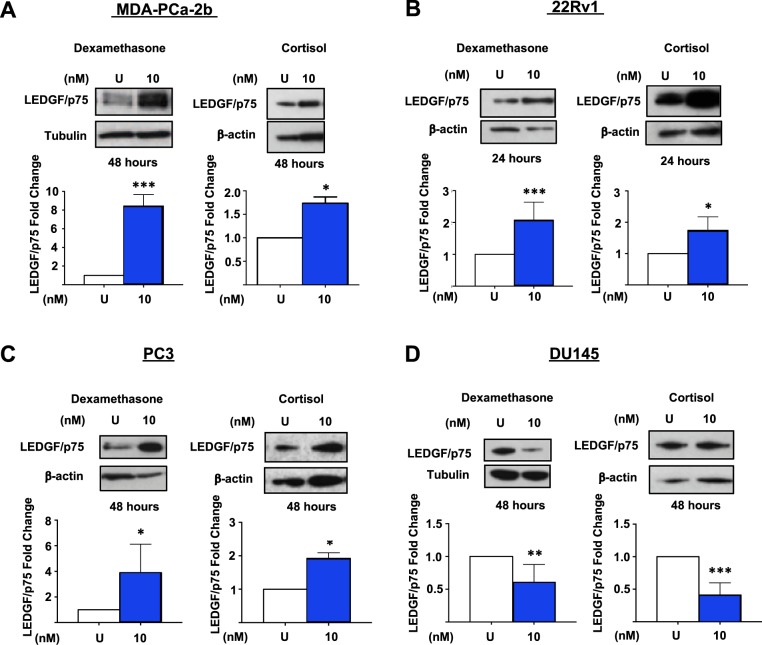
Activated GR signaling upregulates LEDGF/p75 protein expression in PCa cells. Cells were treated every 24 hours with 10 nM Dex or cortisol for up to 48 hours. Untreated (U) cells were used as controls. Whole cell lysates were prepared for Western blotting and probed with anti-LEDGF/p75 antibodies. Results from at least 3 independent experiments were quantified with ImageJ as described in the Methods section. Results reveal increased LEDGF/p75 expression in MDA-PCa-2b **(A)**, 22Rv1 **(B)**, and PC3 **(C)** cells and decreased LEDGF/p75 expression in DU145 **(D)** cells treated with glucocorticoids. Unpaired t-test statistical analysis revealed that LEDGF/p75 induction in MDA-PCa-2b and 22Rv1 cell lines achieved the highest statistical significance (^*^p < 0.05, ^**^p < 0.01, ^***^p < 0.005). All blots represented within panels, although cropped, are derived from the same gel. Full-length blots/gels are presented in Supplementary Figure S2.

We measured the protein expression of LEDGF/p75 in a racially diverse panel of cell lines expressing GR—i.e. MDA-PCa-2b, 22Rv1, PC3, and DU145—following exposure to glucocorticoids, and compared the modulated expression to matched untreated control cells. We treated these cell lines with either 10 nM cortisol or 10 nM dexamethasone (Dex) for up to 48 hours, and collected total lysates of treated and untreated cells for immunoblotting analyses using an antibody specific for LEDGF/p75. We observed increased LEDGF/p75 protein expression in MDA-PCa-2b, 22Rv1, and PC3 cells treated with glucocorticoids (Fig. 1A–C; uncropped Western blot images used to generate Fig. 1 and other figures in this manuscript are shown in Supplementary Fig. S2), with the most robust upregulation observed in MDA-PCa-2b cells and the highest statistical significance achieved in both the MDA-PCa-2b and 22Rv1 cell lines. Both cortisol and Dex treatments led to decreased protein expression of LEDGF/p75 in DU145 cells (Fig. 1D). Interestingly, while 22Rv1 cells upregulated LEDGF/p75 protein expression after 24 hours of glucocorticoid exposure, MDA-PCa-2b, PC3, and DU145 cells required 48 hours of exposure to glucocorticoids to observe significant changes in LEDGF/p75 expression. Initial dose-dependent (0 nM to 100 nM) experiments determined an optimal concentration of 10 nM cortisol and Dex in MDA-PCa-2b and 22Rv1 cells for induction of LEDGF/p75 expression (Supplementary Fig. S3). Dex concentrations in the 10–100 nM range have been commonly used in previous studies to determine *in vitro* GR signaling mechanisms in PCa cells^5,8,16^. We also observed that Dex concentrations higher than 100 nM induced minimal cytotoxicity in a cell type- and time-dependent manner (data not shown). Since we consistently found that Dex treatment induced a more robust modulation of LEDGF/p75 protein expression than cortisol in our PCa cellular models, all subsequent experiments were conducted with Dex, a highly specific and potent GR agonist^84^.

**Figure 2:**
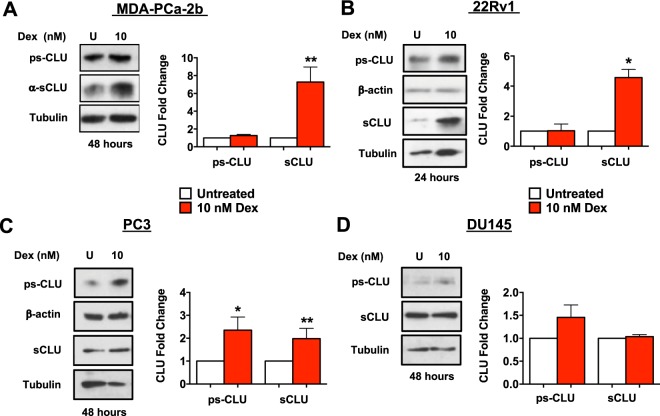
Activated GR signaling upregulates CLU protein expression in PCa cells. Cells were treated every 24 hours with 10 nM Dex for up to 48 hours. Untreated (U) cells were used as controls. Whole cell lysates were prepared for Western blotting and probed with anti-sCLU antibodies. Results from at least 3 independent experiments were quantified with ImageJ as described in the Methods section. Results reveal increased CLU expression in MDA-PCa-2b **(A)**, 22Rv1 **(B)**, and PC3 **(C)** cells treated with Dex. Increased CLU expression was not observed in DU145 **(D)** cells. Unpaired t-test statistical analysis revealed that increase in MDA-PCa-2b and 22Rv1 cell lines achieved the highest fold change compared to untreated controls (^*^p < 0.05, ^**^p < 0.01). All blots represented within panels, although cropped, are derived from CLU and loading control images from the same gel. Full-length blots/gels are presented in Supplementary Figure S2.

Next, we treated our panel of cell lines with 10 nM Dex and collected total lysates of treated and untreated cells to determine the upregulation of CLU using an antibody specific for the α-chain of CLU capable of detecting both psCLU and sCLU. While conducting immunoblotting and exposing the membranes to autoradiography film, we consistently observed a very intense upregulation of sCLU protein in the MDA-PCa-2b and 22Rv1 cell lines compared to the PC3 and DU145 cell lines, which expressed relatively low levels (data not shown). This prevented us from accurately quantifying CLU protein expression levels in these cell lines and comparing them with those in PC3 and DU145. Because of this, in subsequent experiments we loaded only 5 μg of total protein in individual lanes of gels for MDA-PCa-2b and 22Rv1 cells, compared to 20 μg of total protein for PC3 and DU145 cells. After adjusting the protein loading amounts to better match band intensity across cell lines, we continued to observe a statistically significant increase in CLU protein expression in MDA-PCa-2b and 22Rv1 cells, compared to PC3 cells, despite loading 75% less protein in the individual lanes (Fig. 2A–C). In agreement with the results with LEDGF/p75, the most robust upregulation was consistently observed in the MDA-PCa-2b and 22Rv1 cell lines (Fig. 2A,B), whereas treatment of DU145 cells with Dex did not alter the expression of CLU (Fig. 2D).

### LEDGF/p75 and CLU transcript levels increase in response to activated GR signaling

**Figure 3:**
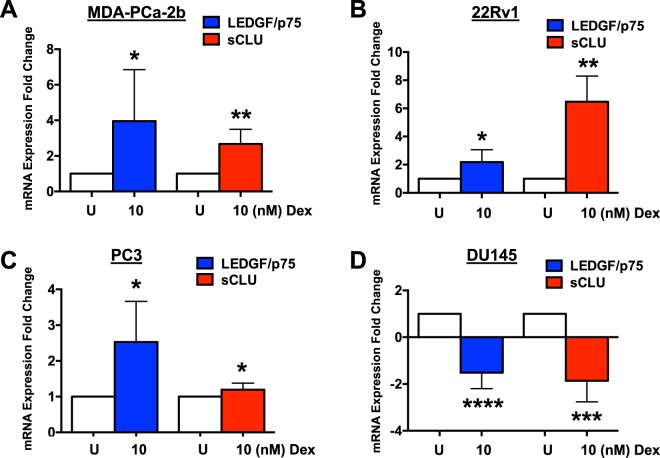
Glucocorticoids increase transcript levels of LEDGF/p75 and CLU in PCa cells. Cells were treated every 24 hours with 10 nM Dex for up to 48 hours. Untreated (U) cells were used as controls. Total RNA was extracted from MDA-PCa-2b, 22Rv1, PC3, and DU145 cells. Results from at least 3 independent qPCR experiments revealed LEDGF/p75 and CLU transcript levels increased in MDA-PCa-2b **(A)**, 22Rv1 **(B)**, and PC3 **(C)** cells exposed to 10 nM Dex. LEDGF/p75 and CLU transcript levels decreased in DU145 **(D)** cells. Unpaired t-test statistical analysis was used to assess statistical significance (^*^p < 0.05, ^***^p < 0.001, ^****^p < 0.0001).

We next determined if the observed elevation in protein expression of LEDGF/p75 and CLU in cells exposed to 10 nM Dex also occurred at the transcript level. For this we conducted qPCR analysis of LEDGF/p75 and CLU transcripts in MDA-PCa-2b, 22Rv1, PC3, and DU145 cells exposed to Dex, using the same experimental conditions described above. Consistent with our immunoblotting results, we observed a significant increase in LEDGF/p75 and CLU transcript expression in MDA-PCa-2b, 22Rv1, and PC3 cells exposed to 10 nM Dex, compared to untreated cells, with significant decrease in transcript expression in DU145 cells (Fig. 3A–D).

### Pharmacological inhibition of GR reduces glucocorticoid-induced protein expression of LEDGF/p75 and CLU in 22Rv1 cells

**Figure 4:**
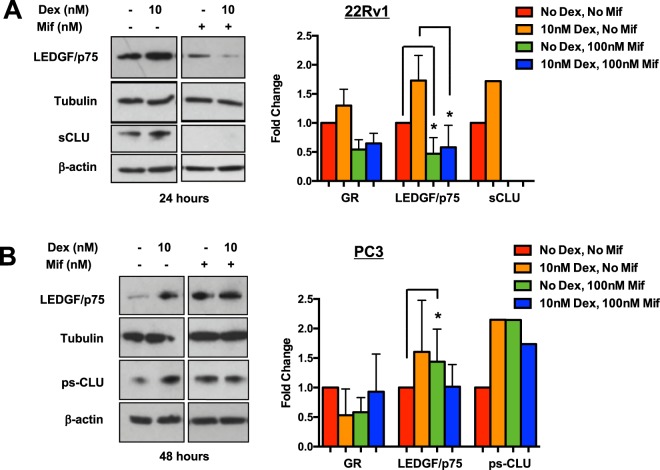
Pharmacological inhibition of GR attenuates glucocorticoid-induced protein expression of LEDGF/p75 and CLU in 22Rv1 cells. 22Rv1 and PC3 cells were co-treated with 10 nM Dex and 100 nM Mif for up to 48 hours. Untreated cells were used as controls. Whole cell lysates were prepared for Western blotting analysis and probed with anti-GR, anti-LEDGF/p75, and anti-sCLU antibodies. Unpaired t-test statistical analysis revealed that Mif attenuated LEDGF/p75 and CLU expression in 22Rv1 cells. **(A)** However, this attenuation was not observed in PC3 cells. **(B)** (^*^p < 0.05) All blots represented within panels, although cropped, are derived from CLU or LEDGF/p75 and loading control images from the same gel. Full-length blots/gels are presented in Supplementary Figure S2.

Given that our initial experiments demonstrated glucocorticoid-induced upregulation of LEDGF/p75 and CLU expression in MDA-PCa-2b, 22Rv1, and PC3 cells, it was necessary to further explore if GR contributes to this upregulation. For these experiments, we inhibited GR function using the GR antagonist mifepristone (Mif). To overcome the inducing effects of 10 nM Dex, we used a concentration of 100 nM Mif. We did not observe cytotoxicity using a 100 nM Mif concentration. We treated 22Rv1 and PC3 cells with 10 nM Dex in the presence or absence of 100 nM Mif in order to compare the effect of GR inhibition on the Dex-induced upregulation of LEDGF/p75 and CLU. Immunoblotting analysis showed that 100 nM Mif attenuated the upregulation of LEDGF/p75 and sCLU protein expression induced by 10 nM Dex in 22Rv1 cells (Fig. 4A). However, this significant attenuation was not observed in PC3 cells under similar experimental conditions (Fig. 4B).

### GR knockdown reduces protein expression of LEDGF/p75 and CLU in PCa cells

**Figure 5:**
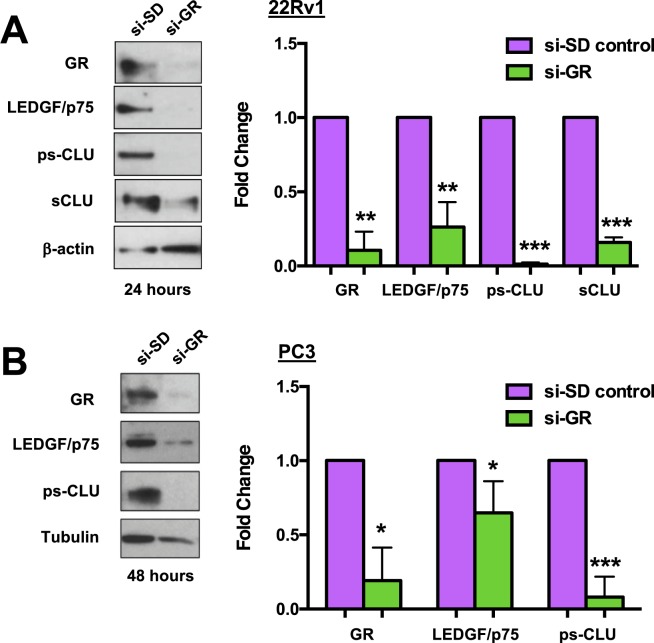
GR knockdown attenuates LEDGF/p75 and CLU protein expression in PCa cells. Transient knockdown of GR was generated in 22Rv1 and PC3 cells (si-GR). Gel electrophoresis using whole lysates of si-GR cells compared to the scrambled control (si-SD control) were probed with anti-GR, anti-LEDGF/p75, and anti-sCLU antibodies. Unpaired t-test statistical analysis revealed that knockdown of GR in 22Rv1 **(A)** and PC3 **(B)** cells attenuated the protein expression of LEDGF/p75 and CLU (^*^p < 0.05, ^**^p < 0.01, ^***^p < 0.005). All blots represented within panels, although cropped, are derived from the same gel. Full-length blots/gels are presented in Supplementary Figure S2.

Recognizing that Mif is not entirely specific for GR since it has been shown by others to be a GR agonist under certain conditions^85^, we sought to specifically target GR to determine if it plays a more direct role in the glucocorticoid induction of LEDGF/p75 and CLU. For these experiments we transiently knocked down GR using a pool of short inhibitory RNAs (siRNAs) specific for GR in 22Rv1 and PC3 cells. These siRNAs target the Nuclear Receptor Subfamily 3 Group C Member 1 (*NR3C1)* gene, which encodes GR. Transient knockdown of GR with siRNAs (si-GR) in 22Rv1 and PC3 cells led to significant depletion of LEDGF/p75 and CLU proteins compared to cells transfected with scrambled duplex siRNA (si-SD) control (Fig. 5A,B). Taken together, these results suggested a direct contribution of GR to glucocorticoid-induced upregulation of LEDGF/p75 and CLU in PCa cells.

### Glucocorticoid signaling modulates PCa cell migration

**Figure 6:**
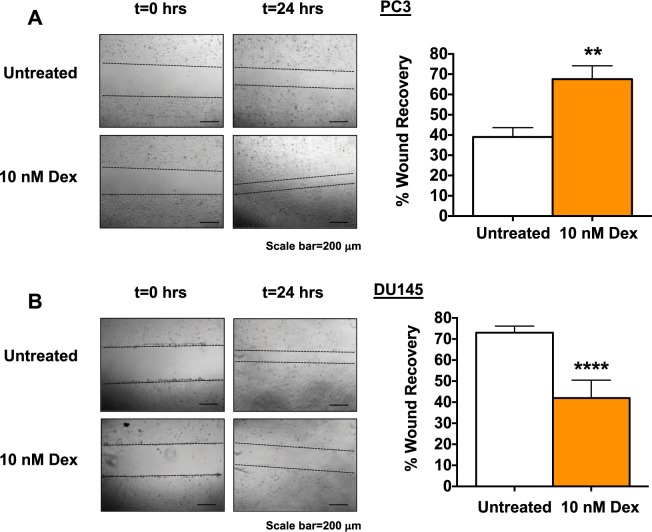
Dexamethasone modulates PCa cell migration. Phase contrast images of scratch wound healing assays used to measure the migration of PC3 and DU145 cells were captured with an Olympus IX70 microscope. PC3 and DU145 cells treated with 10 nM Dex or untreated controls at 0 and 24 hours after the scratches were made at the same point. Unpaired t-test statistical analysis revealed that treatment with 10 nM Dex increases PC3 cell migration **(A)** but decreases DU145 cell migration **(B)**. Scale bar = 200 µm; (^**^p < 0.01, ^****^p < 0.0001).

Glucocorticoid treatment as well as LEDGF/p75 depletion have been shown previously to decrease the migration of DU145 cells and breast cancer cells^46,48,86^. This led us to examine the ability of glucocorticoids to alter the migration rate of PC3 and DU145 cells using scratch wound healing assays. We chose these two cell lines because of their ability to form a confluent monolayer, in contrast with the MDA-PC-2b and 22RV1 cell lines, which typically grow in clusters. After creating a wound area and treating cells with 10 nM Dex, we captured images of the cell cultures at 0 hours and 24 hours in order to track cell migration in treated versus untreated cells. We observed a marked and significant increase in the rate of migration of Dex-treated PC3 cells, compared to untreated cells, such that closure of the wound area occurred at 24 hours (Fig. 6A). However, the opposite effect was observed in DU145 cells, as the rate of migration was significantly reduced in Dex-treated cells compared to untreated cells (Fig. 6B).

### GR binding sites identified in promoter regions of LEDGF/p75 and CLU

**Figure 7:**
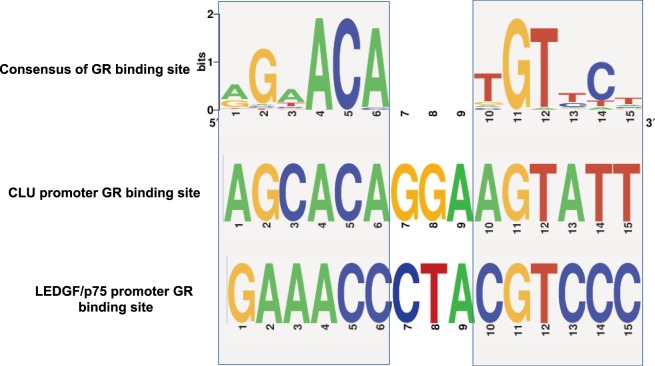
Predicted GR binding sites in LEDGF/p75 and CLU promoter regions. The GR binding consensus (top) derived from experimentally validated sites is aligned with the putative GR binding sites detected within the promoters of CLU (middle) and LEDGF/p75 (bottom). Light gray shaded boxes are used to indicate the conserved half-sites in CLU and LEDGF/p75. GR binding site logos were created using WebLogo.

To further explore the possibility that GR regulates the glucocorticoid induction of LEDGF/p75 and CLU in PCa cells, likely by binding to the promoter regions of these genes, we conducted a PubMed literature survey of GR promoter binding sites as well as an *in-silico* promoter analysis. These resulted in a comprehensive list of GR binding sites (Supplementary Table S1) that have already been experimentally validated. The 49 previously identified GR binding sites ranged from 7 to 35 bases in length (Supplementary Table S1), with 30 of these being 15 bases long. We used the sequences of these 30 GR binding sites to build a consensus GR binding element using WebLogo (Fig. 7).

From the consensus sequence, it could be derived that the GR binding site in most cases consists of two half-sites, which are six base inverted repeats, separated by 3 degenerate bases (Fig. 7). We observed that the last three bases (4–6) of the first (5′) half-site and the first three bases (10–12) of the second (3′) half-site are highly conserved (Fig. 7). To determine if a similar 15 base long putative GR binding site is present in the promoters of LEDGF/p75 and CLU, we scanned these promoters with a letter probability matrix (Supplementary Table S2) derived from their sequences, using FIMO^87^, a webtool, within the MEME Suite 4.12.0. Putative GR binding sites were detected in both CLU (AGCACAGGAAGTATT; *P* < 0.0001) and LEDGF/p75 (GAAACCCTACGTCCC; *P* < 0.0008) promoters, in the reverse strands between 132–146 bases and 611–625 bases, respectively (Fig. 7; Supplementary Table S3).

We also observed from the list of experimentally validated GR-binding sites (Supplementary Table S1), that a half-site is sufficient to bind GRs and the two half-sites may sometimes be separated by four instead of three bases^88–90^. Taking these into consideration we scanned the two promoters for the half-sites with the help of an in-house PERL script and identified at least two half-sites in each of the two promoters (Supplementary Table S3). This *in-silico* analysis suggested that there are multiple putative GR binding sites within the LEDGF/p75 and CLU promoters to which GR might bind and regulate the expression of these genes.

### Analysis of cancer gene microarray datasets reveals race/ethnicity-related differential expression of GR transcript in PCa tissues

**Figure 8:**
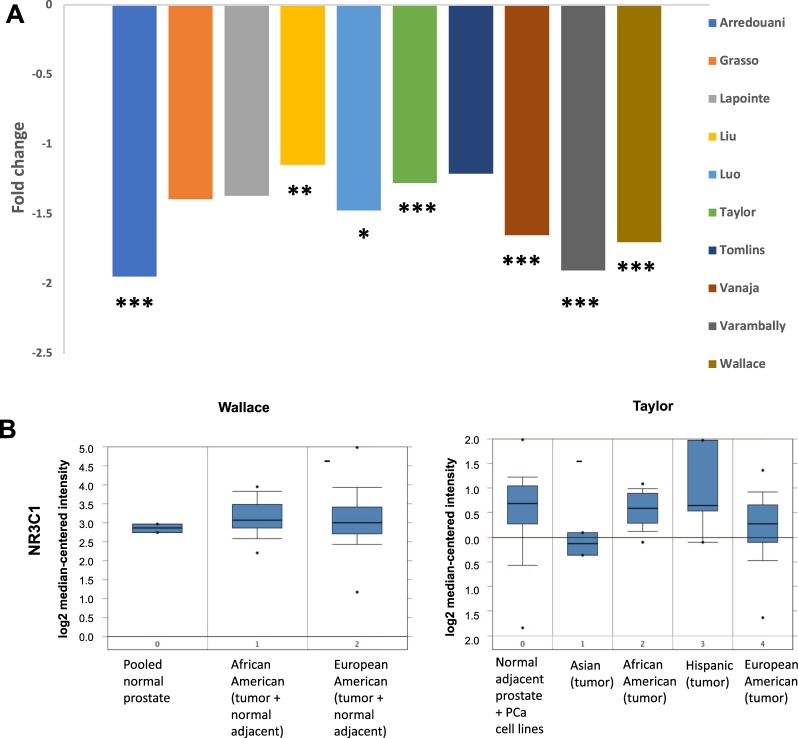
Expression of GR/NR3C1 transcript between clinical prostate cancer tissues specimens from African Americans and European Americans in Oncomine. Transcript expression levels of GR/NR3C1 in prostate tumors versus normal prostate tissues was derived from cancer gene microarray datasets in Oncomine database. Individual dataset names appear in the legend boxes at the right (**A**). Box-plots from the Wallace and Taylor datasets were used from Oncomine gene microarray database (**B**). Fold-changes and corresponding *P-*values for the differences in gene expression between PCa and normal prostate tissues were obtained from Oncomine. The number of samples in each dataset is different, therefore higher fold change does not always correspond to statistical significance. ^*^P < 0.05, ^**^P < 0.01, ^***^P < 0.001.

Next, we sought to examine GR expression (*NR3C1* gene) in human PCa tissues. Transcript expression of *NR3C1* in PCa tissues, compared to normal prostate tissue, was analyzed using 10 PCa gene microarray datasets from the Oncomine database. *NR3C1* was consistently downregulated in prostate tumors compared to normal prostate tissues in 9 of the 10 datasets (Fig. 8A). The magnitude of the fold-decrease was modest, with only 5 datasets showing >1.5-fold downregulation and the remaining 4 datasets with >1-fold decrease. However, the *P* values were <0.001 in 7 datasets, indicating that GR downregulation was highly significant in PCa tissues compared to normal tissues.

Most of the Oncomine datasets examined above have no racial/ethnic identifiers. However, two datasets, Wallace and Taylor, had gene expression data for PCa tissues from different racial/ethnic groups. When grouping the *NR3C1* transcript expression data by race/ethnicity, box-plots of the log2 median-centered intensity in the Wallace dataset showed that the median value was slightly higher in prostate tissues from AA men (3.065) as compared to EA men (2.993) (Fig. 8B). The Taylor dataset displayed a much higher log2 median-centered intensity *NR3C1* transcript in AA (0.589) PCa tissues compared to EA (0.272) and Asian (−0.134) PCa tissues. Interestingly, the *NR3C1* median expression levels in AA PCa tissues was slightly lower than that in PCa tissues from Hispanic patients (0.64) in the Taylor dataset. Unfortunately, we did not have access to racial identifiers for the Hispanic patients. It is noteworthy that the Wallace dataset grouped the normal adjacent prostate tissues with prostate tumors for the race/ethnicity-based analysis, while the Taylor dataset grouped the normal adjacent and PCa tissues separately, which may account for the differences in *NR3C1* expression between AA and EA PCa tissues observed in these two datasets.

## Discussion

Understanding the mechanism(s) by which GR signaling activates AR-target genes in the absence of androgens has high relevance to the development of effective treatments for advanced PCa^2,5–10^. The downstream effects of this GR bypass include promotion of tumor aggressiveness as well as resistance to standard PCa therapies such as ADT and potentially taxane chemotherapy^5,7,16^. While key stress oncoproteins associated with increased PCa cell survival in the presence of environmental stressors such as chemotherapeutic drugs have been identified and at times targeted in pre-clinical and clinical studies (e.g., CLU, LEDGF/p75, HSP27, PRDXs), there remains a need to understand the potential contribution of glucocorticoid signaling to the activation and upregulation of these proteins. With this goal in mind, this study was designed to examine the contribution of glucocorticoid signaling to the expression of CLU and LEDGF/p75, two stress oncoproteins previously established as key contributors to therapy resistance in various cancer types^18,20–22,26–29^. The roles of CLU and LEDGF/p75 in the acquisition and maintenance of resistance to standard PCa therapies have been established; however, this study is the first to implicate GR signaling in their upregulation in PCa cells. Multiple recent studies have linked GR signaling to PCa disease progression^2,5,7–10,16,91^, but to our knowledge none of these studies have compared GR signaling events in racially diverse pre-clinical cellular models, or explored GR expression in racially diverse PCa tissues.

In this study, we observed that exposure to glucocorticoids led to upregulation of CLU and LEDGF/p75 transcript and protein levels in three out of four PCa cell lines, and that GR blockade attenuated this effect. Our finding that CLU expression is highly responsive to glucocorticoids in MDA-PCa-2b and 22Rv1 cells is underscored by our decision to decrease substantially the amount of total protein from these two cell lines that were loaded in individual lanes of gels for immunoblotting. For instance, when we loaded 20 μg of protein per lane from MDA-PCa-2b and 22Rv1 cells, CLU protein expression was so dramatically elevated that we were unable to differentiate individual lanes within the blots, even after decreasing the exposure time. Conversely, CLU protein expression was relatively lower in PC3 and DU145 cells when individual lanes in gels were loaded with 20 μg of protein from these cells. The observed high endogenous expression of CLU in the MDA-PCa-2b cell line, derived from an AA patient, could suggest that downstream effects of GR signaling such as the upregulation of therapy-resistance associated genes may be exaggerated in AA PCa patients. This would be consistent with the emerging notion that GR signaling is enhanced in the AA population^52,63^.

Interestingly, we observed downregulation of LEDGF/p75 and CLU in DU145 cells exposed to glucocorticoids, which was consistently opposite from the results observed in MDA-PCa-2b, 22Rv1, and PC3 cells. This was expected as other investigators have reported that GR activation decreases the aggressive properties of the DU145 cell line while its blockade reverses this effect^84,92–94^. The impact of activated GR signaling on PCa cells includes the promotion of tumor aggressiveness properties leading to worse overall patient survival^5,8,16,91^. Tumor aggressiveness develops when normal cellular functions are altered and established hallmarks of cancer such as increased cell migration are induced^95^. We observed that Dex treatment significantly increased the migration rate in PC3 cells but not that of DU145 cells, suggesting a cell-type dependent effect. These results are consistent with our observation that GR induced downregulation of LEDGF/p75 in DU145 cells, and previous reports that LEDGF/p75 depletion or treatment with Dex decreased the migration rate of DU145 PCa cells^47,86^. Several potential mechanisms have been proposed to explain these DU145-specific effects, including relatively higher GR expression compared to other PCa cell lines, which could lead to immediate downregulation of GR and shutdown of GR signaling upon exposure to glucocorticoids^84,94^.

The ability of glucocorticoids to upregulate LEDGF/p75 and CLU in PCa cell lines appears to be mediated by GR. This was confirmed by our observation that pharmacological or genetic blockade of GR led to significant downregulation of LEDGF/p75 and CLU expression. Although it remains to be established in follow-up studies that GR binds directly to and activates promoter regions of these two genes, our *in-silico* analysis confirmed the presence of multiple putative GR binding sites within both LEDGF/p75 and CLU promoter regions, which would allow for direct regulation by activated GR. This would be consistent with recent observations that DEX-induced GR activation is associated with DTX resistance in PCa and breast cancer cells^17,18^.

Recent studies have demonstrated that GR expression is reduced in primary PCa tissues but increases in metastatic lesions, particularly in patients who have received DTX therapy^7,16^. Since these analyses were conducted mostly in PCa tissues from EA patients, we asked whether primary PCa tissues from AA men also express reduced levels of GR. Our analysis of 10 cancer gene microarray datasets from the Oncomine database revealed that GR (encoded by *NR3C1* gene) transcript was consistently downregulated in prostate tumors compared to normal prostate tissues, in agreement with the previous reports^7,16^. However, when we focused our analysis on GR expression based on racial classification, we observed differences between AA and EA PCa patients. Both the Wallace and Taylor datasets, which contain gene expression data from AA PCa tissues, revealed higher median values of GR in AA prostate tissues compared to EA prostate tissues. Unfortunately, there was no information in the datasets regarding the chemotherapy treatment status of the tissue donors. Nevertheless, these findings are consistent with the premise that AA men with PCa may have enhanced intratumoral GR signaling.

We speculate that the recently documented hyperactive GR signaling occurring in AA men^52,63^ could exacerbate the upregulation of LEDGF/p75 and CLU in PCa cells. It is possible that chronically elevated cortisol levels, increased GR levels, and hyperactive GR signaling sustained in AA men over time could prime them to develop aggressive PCa tumors. In addition, this enhanced GR signaling could induce a robust expression of oncoproteins associated with therapy-resistance, including LEDGF/p75 and CLU, leading to poor response to conventional treatments in AA PCa patients.

The results of this study also complement the growing body of literature suggesting that glucocorticoid co-administration with PCa therapies including ADT may potentially lead to worse overall patient survival^2,5,7,10–12^. By upregulating oncoproteins associated with resistance to ADT and taxane chemotherapy, activated GR signaling may promote the proliferation and migration of highly aggressive PCa tumor cells with enhanced therapy resistance capabilities. For instance, our findings that GR activation increased CLU could offer insights into why recent clinical trials targeting CLU with an antisense oligonucleotide-based drug, Custersin, in combination with taxane chemotherapy in advanced stage PCa patients were ineffective^96,97^. It is plausible that CLU and other pro-survival proteins were activated by GR as patients in both study arms received glucocorticoid co-therapy to mitigate side effects.

The implications of our findings are far-reaching as GR is emerging as a key driver of PCa tumor aggressiveness, especially in the absence of AR signaling. Since our results demonstrate that LEDGF/p75 and CLU are upregulated in the absence of androgen via GR, future combinatorial therapies co-targeting AR, GR, and stress oncoproteins could potentially confer greater overall survival to patients with advanced PCa. In addition, given that AA men display an enhanced physiological response to glucocorticoids as well as disproportionate PCa incidence and mortality, further studies are needed to better elucidate the relationship between GR signaling and PCa tumor aggressiveness specifically in this racial/ethnic group.

## Methods

### Cell lines, antibodies, and reagents

All cell lines were purchased from the American Type Culture Collection (ATCC) and grown in a humidified incubator with 5% CO_2_ at 37°. In accordance with recent guidelines by the NIH on authentication of key biological resources, cells were authenticated utilizing Short Tandem Repeat (STR) profiling against the ATCC STR database (ATCC, Cat: ATCC 135-XV). Cells were routinely tested for mycoplasma contamination using MycoAlert™ PLUS Mycoplasma Detection Kit (Lonza, Cat:LT07). PC3 (Cat:CRL-1435), DU145 (Cat:HTB-81), and 22Rv1 (Cat:CRL-2505) cell lines were cultured in RPMI 1640 medium (Corning, Cat:10-040-CV) supplemented with 10% fetal bovine serum (Corning, Cat:35010CV), penicillin-streptomycin (Corning, Cat:30001Cl), and gentamicin (Gibco, Cat:15710064) as recommended by the supplier. MDA-PCa-2b (Cat:CRL-2422) cell line was cultured in F-12K medium (ATCC^®^, Cat:30–2004) supplemented with 20% fetal bovine serum (Corning, Cat:35010CV), cholera toxin (Sigma-Aldrich, Cat:C8052), epidermal growth factor (Sigma-Aldrich, Cat:E4127), *o*-phosphoethanolamine (Sigma-Aldrich, Cat:P0503), hydrocortisone (Sigma-Aldrich, Cat:H0888), selenious acid (ACROS Organics, Cat:AC19887), bovine insulin (Sigma-Aldrich, Cat:I6634), and penicillin-streptomycin as recommended by the supplier. 0.2% Normocin (Invivogen, Cat:ANT-NR-1) was added to the medium for PC3, DU145, 22Rv1, and MDA-PCa-2b cell lines to prevent contamination by mycoplasma, bacteria, or fungi.

Cortisol (Sigma-Aldrich, Cat:H0888) and Dex (Sigma-Aldrich, Cat:D4902) reconstituted in ethanol were used at 10 nM concentrations as GR agonists while Mif (Sigma-Aldrich, Cat:M8046) reconstituted in ethanol was used at 100 nM concentration as a GR antagonist. Initial dose-dependent (0 nM to 100 nM) experiments determined an optimal concentration of 10 nM cortisol and Dex. DHT (Cat: D073) reconstituted in ethanol was used at 1 nM and 10 nM concentrations as AR antagonists while Enz (HY-70002 Medchem Express) reconstituted in ethanol was used at 1 μM as an AR antagonist. In all experiments incorporating cortisol, Dex, Mif, DHT, or Enz, charcoal-stripped fetal bovine serum (Atlanta Biologicals, Cat:S11650) was used in order to selectively remove hormones while avoiding non-specific loss of other serum components. For experiments exceeding 24 hours, medium was replaced every 24 hours to ensure that cells would have consistent exposure to glucocorticoids. During the optimization of each experiment, vehicle (ethanol) was included for every untreated control.

The following commercially-acquired antibodies were used: rabbit polyclonal anti-LEDGF/p75 (1:1000, Bethyl Laboratories Inc., Cat:A300-848A), mouse monoclonal anti-GR (1:1000, BD Biosciences, Cat:611226), mouse monoclonal anti-clusterin α-chain (1:1000, Millipore, Cat:05–354), rabbit monoclonal anti-AR (1:1000, Cell Signaling, Cat: 5153 S), rabbit monoclonal anti-β-actin (1:5000, Cell Signaling, Cat:5125), rabbit polyclonal anti-α/β-tubulin (1:1000, Cell Signaling, Cat:2148 S).

### Immunoblotting procedures

Immunoblotting was performed as described previously^41^. Briefly, equal amounts of protein from whole cell lysates (5 μg for MDA-PCa-2b and 22Rv1; 20 μg for PC3 and DU145) were separated using sodium dodecyl sulfate polyacrylamide gel electrophoresis (SDS-PAGE, NuPAGE 4–12%, Thermo Fisher Scientific, Cat:NP0321BOX) and transferred into polyvinyl difluoride membranes (Millipore, Cat:IPFL00010). Membranes were blocked in 5% dry milk prepared in TBS-T buffer (20 mM Tris- HCl, pH 7.6, 140 mM NaCl, 0.1% Tween 20). Membranes were then probed individually with primary antibodies and corresponding secondary antibodies and washed several times with TBS-T between each antibody application. Enhanced chemiluminescence (ECL) was used to detect immunoreactive protein bands. For this, the ECL Western Blotting Substrate (Thermo Fisher Scientific Pierce, Cat:32106) was added to the antibody-protein surface of each membrane, followed by incubation for 4 minutes. Membranes were then transferred to autoradiography cassettes and exposed to autoradiography films for different lengths of time to ensure accurate detection of immunoreactive protein bands. Protein bands from at least 3 independent experiments for each treatment were quantified using ImageJ Software. The ratios of CLU or LEDGF/p75 protein bands to the loading control bands (tubulin or β-actin) were normalized to one in control, untreated samples. This was then used to calculate the fold-upregulation of CLU and LEDGF/p75 in the treated samples. Depending on the cell line, we used either tubulin or β-actin as loading controls since we observed in initial experiments that glucocorticoids induced β-actin in a cell line-dependent manner.

### Quantitative real-time PCR

Quantitative Real-Time PCR (qPCR) was performed as described previously^41^. Briefly, Total RNA was extracted from cells using the RNeasy Plus Mini Kit (Qiagen, Cat:74134). The iScript cDNA synthesis kit (BioRad, Cat:1708891) was used to reverse transcribe RNA (0.5 μg) into cDNA. qPCR was performed using the MyiQ real-time PCR detection system with primers using iQ SYBR Green Supermix (BioRad, Cat:1708882) following manufacturers’ recommendations. Primer sequences for LEDGF/p75, Clusterin, and glyceraldehyde 3-phosphate dehydrogenase (GAPDH) were designed using Primer 3 software. The forward sequence for LEDGF (5′ to 3′) was TGCTTTTCCAGACATGGTTGT and reverse sequence (5′ to 3′) was CCCACAAACAGTGAAAAGACAG. The forward sequence for Clusterin (5′ to 3′) was CTCTACTCTCCGAAGGGAATTGTC and reverse sequence (5′ to 3′) was CGGGCTGCCTGTGCAT. GAPDH mRNA was used for normalization and the forward sequence (5′ to 3′) was GAGTCAACGGATTTGGTCGT and reverse sequence (5′ to 3′) was TTGATTTTGGAGGGATCTCG. Primers were commercially synthesized by Integrated DNA Technologies (IDT). Data was normalized to values of corresponding untreated controls and analyzed in at least three independent experiments, each in triplicates.

### RNA interference-mediated knockdown of GR in PCa cells

To achieve transient knockdown of GR in our cellular models, commercially-available specific short inhibitory Trisilencer-27 RNAs (Origene, Cat:SR301960) corresponding to Locus ID: 2908 were used as described previously^43^. Cells were transfected in a pooled knockdown with 10 nM of each siRNA Trisilencer-27/Dicer-Substrate duplex using oligofectamine (Invitrogen, Cat:12252011) following manufacturer’s instruction. A universal scrambled negative control siRNA duplex (Origene, Cat:SR30004) was used as a negative control. Quantification of protein bands in immunoblots was performed as indicated above.

### Cell migration

In order to evaluate the migratory response of PC3 and DU145 cells following exposure to Dex, a scratch wound healing assay was performed. Cells were seeded to confluency and grown for 24 hours in RPMI 1640 medium supplemented with 10% charcoal-stripped FBS, with or without 10 nM dexamethasone. Wound areas were generated using a 200 µl pipette tip by scratching the cell surface confluent monolayer. Migration of cells into the wound areas was visually tracked using an Olympus IX70 microscope equipped with SPOT RT3 Imaging System and phase contrast images were captured at 0 and 24 hours. The wound recovery rate of migrating cells obtained in 6 independent experiments was measured using ImageJ Software. The wound area at 24 hours was compared to the wound area at 0 hours within each group to determine the % wound recovery.

### Identification of GR binding sites

The predicted consensus GR binding sites in CLU and LEDGF/p75 promoters were created using WebLogo^98^. The 30 sequences (15 base long GR binding sites) used as input are shown in Supplementary Table S1. The same sequences were used to create a letter probability matrix (Supplementary Table S2). The CLU and LEDGF/p75 promoters were scanned for putative GR binding sites with this matrix, using FIMO within the MEME Suite 4.12.0. For the identification of GR binding half sites a PERL script was used that was developed in-house. Promoters were searched for 6 base long half sites with appropriate degeneracies, the search was then expanded on either the 5′ or the 3′ end depending upon the half-site that was detected. Logos for the putative GR binding motifs (Fig. 7) were represented according to existing guidelines^99^.

### Bioinformatics analysis of oncomine cancer gene microarray database

For analysis of mRNA expression of GR, encoded by the *NR3C1* gene, in PCa and normal prostate tissues, we selected 10 datasets from the Oncomine database (Compendia Biosciences; Ann Arbor, MI; www.oncomine.org). These datasets, derived from gene microarray analyses of PCa and normal prostate tissues, provide fold-change data for gene expression with *P* values calculated by Oncomine using Student’s *t*-tests. Two out of the 10 datasets, Wallace and Taylor, included ethnicity/race data. The Wallace dataset included 41 prostate tissue specimens from AA men and 46 from EA men while the Taylor dataset included 24 AA PCa and 115 PCa EA specimens in addition to 2 Asian and 5 Hispanic PCa tissue samples. 29 normal adjacent tissue samples were racially pooled. This allowed us to compare the *NR3C1* transcript expression between tissues from AA and EA patients.

### Statistical analysis

SPSS Statistics Software V22.0 and GraphPad Prism 6 were used for statistical analyses. Differences between treatment groups were analyzed using unpaired Student’s t-test. P values below 0.05 were considered statistically significant. All differences highlighted by asterisks were statistically significant as encoded in figure legends (*P < 0.05; **P < 0.01; ***P < 0.001; ****P < 0.0001).

## Electronic supplementary material

Supplementary Information

## Data Availability

All data generated or analyzed during this study are available from the corresponding author upon reasonable request.
