# Supplementary material for: Glucocorticoids Induce Stress Oncoproteins Associated with Therapy-Resistance in African American and European American Prostate Cancer Cells
Source: Sci Rep. 2018 Oct 10;8:15063. doi: 10.1038/s41598-018-33150-2 (PMC6180116; doi:10.1038/s41598-018-33150-2)
Supplement: Supplementary file 1 — Supplementary Information [file 41598_2018_33150_MOESM1_ESM.pdf]

Glucocorticoids Induce Stress Oncoproteins Associated with Therapy-Resistance in African American and European American Prostate Cancer Cells

[1]Leanne Woods-Burnham, [1]Christina K. Cajigas-Du Ross, [1]Arthur Love, [1]Anamika Basu, [1]Evelyn S. Sanchez-Hernandez, [1]Shannalee R. Martinez, [1]Greisha L. Ortiz-Hernández, [2]Laura Stiel, [1]Alfonso M. Durán, [3]Colwick Wilson, [2]Susanne Montgomery, [4]Sourav Roy, and [1,5]Carlos A. Casiano

[1]Center for Health Disparities and Molecular Medicine and Department of Basic Sciences, Loma Linda University School of Medicine, Loma Linda, CA, USA; [2]Loma Linda University School of Behavioral Health, Loma Linda, CA, USA; [3]Oakwood University, Huntsville, AL, USA; [4]Department of Entomology and Institute for Integrative Genome Biology, University of California Riverside, Riverside, CA, USA; [5]Department of Medicine, Loma Linda University School of Medicine, Loma Linda, CA, USA.

Author for correspondence: Carlos A. Casiano, Ph.D., Center for Health Disparities and Molecular Medicine, Mortensen Hall 142, 11085 Campus St., Loma Linda University School of Medicine, Loma Linda, CA 92350, USA; Tel: 1-909-558-1000 ext. 42759; Fax: 1-909-558-0196; Email: ccasiano@llu.edu

Supplementary Figure S1

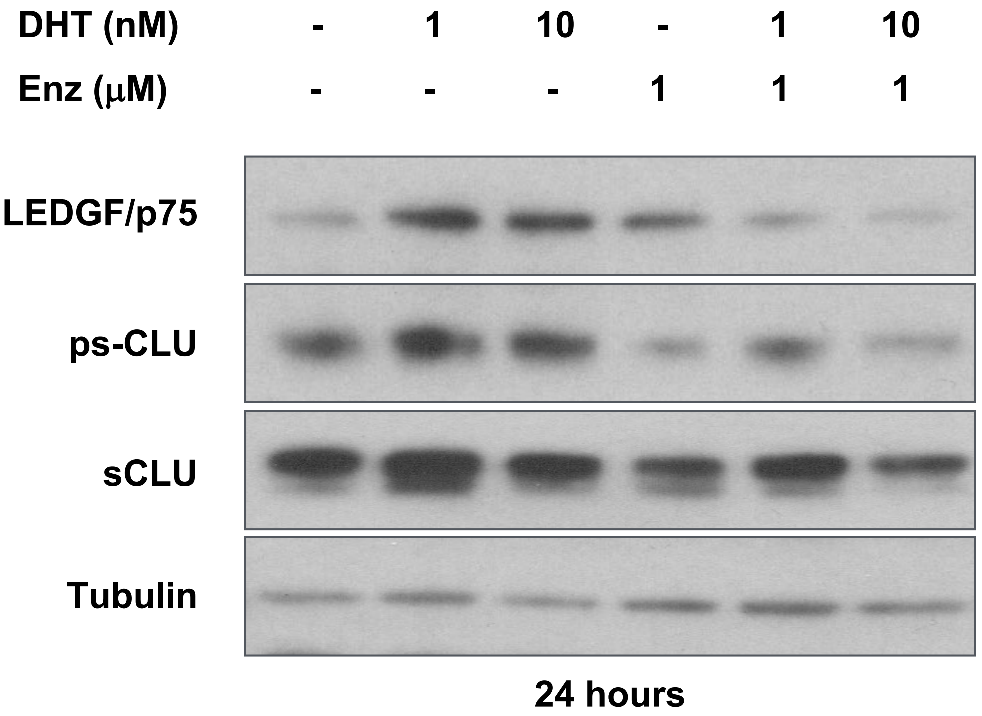

Supplementary Figure S1. Androgen-induced upregulation of LEDGF/p75 and CLU in 22Rv1 cells. Cells were treated for 24 hours with DHT or Enz. Untreated (U) cells were used as controls. Whole cell lysates were prepared for Western blotting and probed with anti-LEDGF/p75 and anti-sCLU antibodies. Results from two independent experiments were quantified with ImageJ. Results reveal a trend of increased LEDGF/p75 and CLU expression in 22Rv1 cells treated with DHT. Enz attenuated LEDGF/p75 and CLU expression. This image is uncropped and all bands are derived from the same gel.

Supplementary Figure S2

Uncropped Gel Images

Full Scan for Fig 1A-Dexamethasone

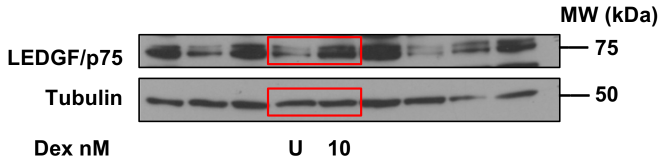

Full Scan for Fig 1B-Dexamethasone

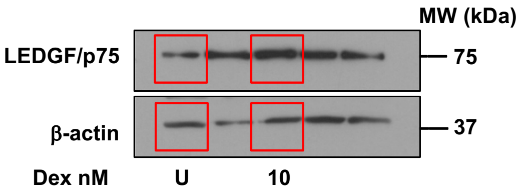

Full Scan for Fig 1A-Cortisol

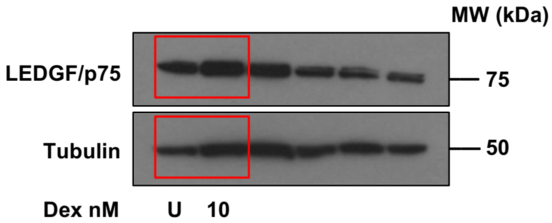

Full Scan for Fig 1B-Cortisol

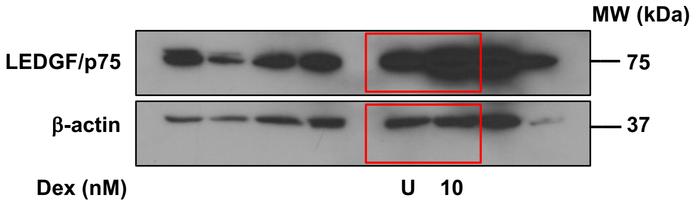

Supplementary Figure S2. Complete representation of full-length blots/gels. All western blot images shown in this manuscript are provided in full-length without cropping.

Supplementary Figure S2 (cont.)

Full Scan for Fig 1C-Dexamethasone

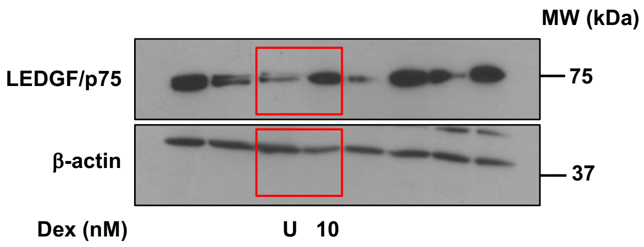

Full Scan for Fig 1D-Dexamethasone

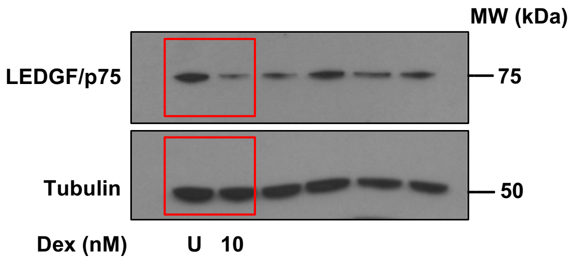

Full Scan for Fig 1C-Cortisol

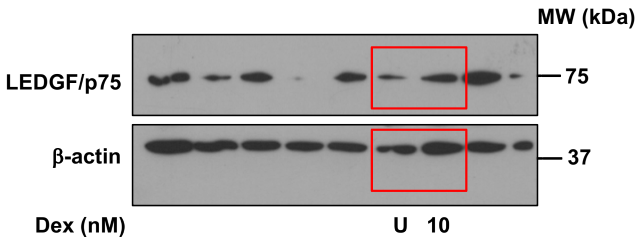

Full Scan for Fig 1D-Cortisol

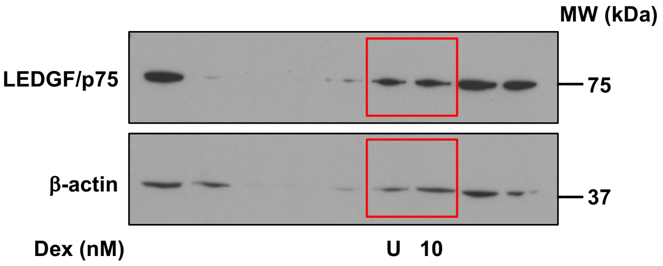

Supplementary Figure S2 (cont.)

Full Scan for Fig 2A

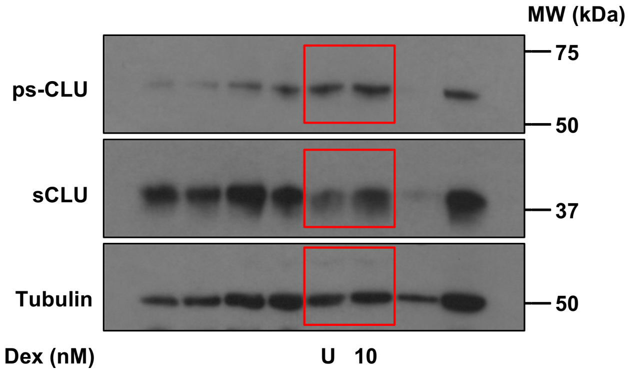

Full Scan for Fig 2B

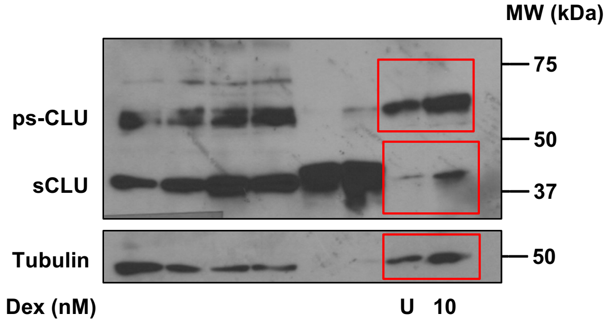

Full Scan for Fig 2C

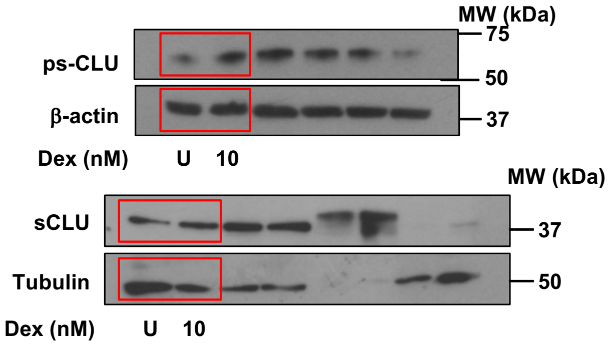

Full Scan for Fig 2D

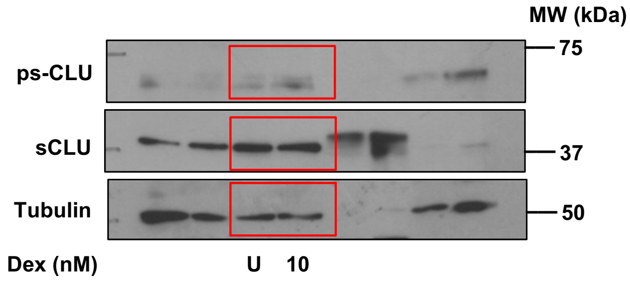

Supplementary Figure S2 (cont.)

Full Scan for Fig 4A

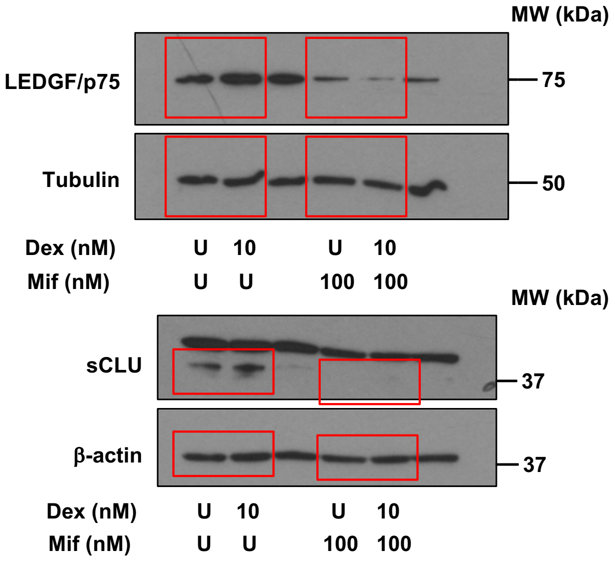

Full Scan for Fig 4B

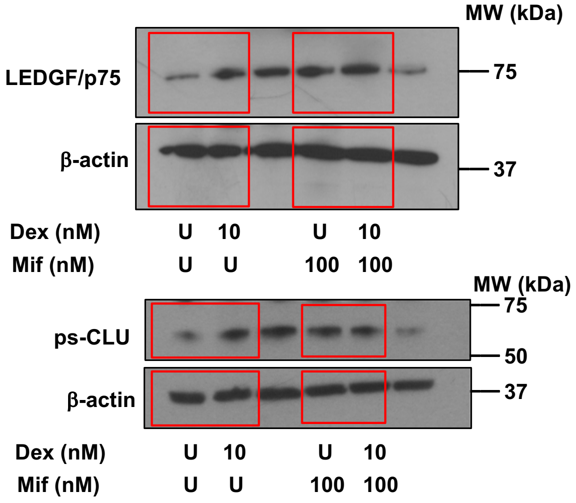

Supplementary Figure S2 (cont.)

Full Scan for Fig 5A

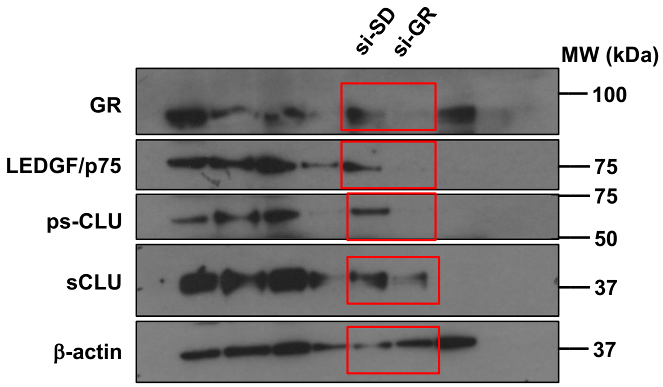

Full Scan for Fig 5B

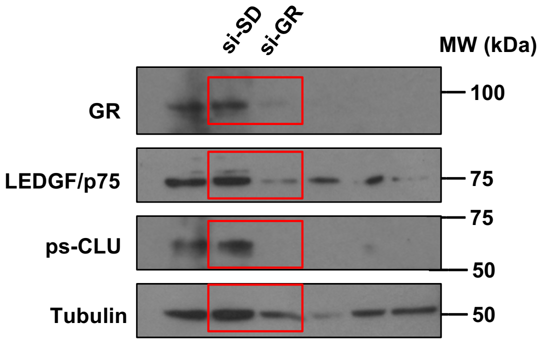

Supplementary Figure S3

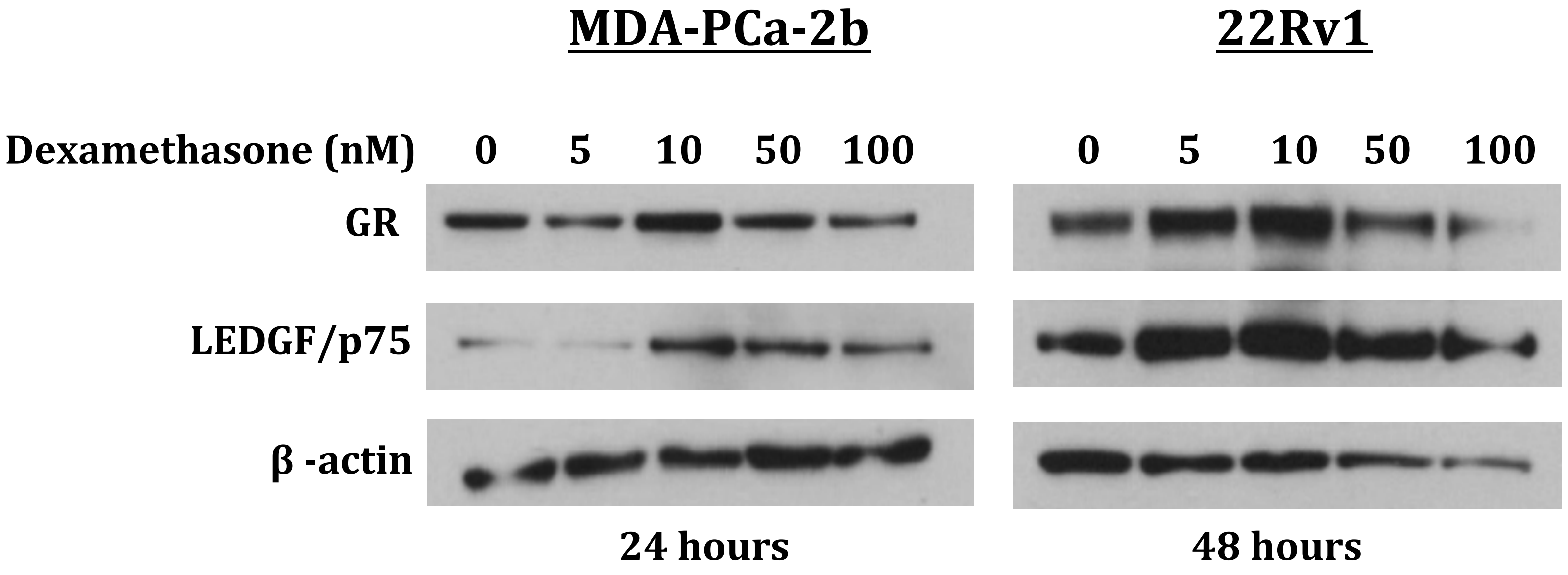

Supplementary Figure S3. Dose-dependent treatment of MDA-PCa-2b and 22Rv1 cells with Dex. Cells were initially treated in a dose-dependent manner with Dex. Whole cell lysates were prepared for Western blotting and probed with anti-GR and anti-LEDGF/p75 antibodies. Results revealed the most robust upregulation of GR and LEDGF/p75 protein expression in MDA-PCa-2b and 22Rv1 cells treated with 10 nM Dex. This image is uncropped and all bands are derived from the same gel.

Supplementary Table S1

Gene	Site	Size (bp)	Source
GilZ	AGAACAttgGGTTCC	15	(Sebastiaan H. Meijsing et al. 2009)
GilZ	GGAACCCAATGTTCT	15	(Jen-Chywan Wang et al. 2004)
GilZ	TTAACAGAATGTCCT	15	(Jen-Chywan Wang et al. 2004)
GilZ	CAAACACCGTGTTCA	15	(Jen-Chywan Wang et al. 2004)
GilZ	CCTGCACCTTTGTTCT	16	(Jen-Chywan Wang et al. 2004)
Pal	AGAACAaaaTGTTCT	15	(Sebastiaan H. Meijsing et al. 2009)
Sgk	AGAACAtttTGTCCG	15	(Sebastiaan H. Meijsing et al. 2009)
Tat	AGAACAtcccTGTACA	16	(Sebastiaan H. Meijsing et al. 2009)
Cgt	AGAACAtttTGTACG	15	(Sebastiaan H. Meijsing et al. 2009)
Cons	AGAACAaaaTGTACC	15	(Sebastiaan H. Meijsing et al. 2009)
FKBP5	AGAACAgggTGTTCT	15	(Sebastiaan H. Meijsing et al. 2009)
hKLF15GRE1	caattacatgttgttcctg	19	(Maki Asada et al. 2011)
hKLF15GRE2	ccggaacagcgtgtcccct	19	(Maki Asada et al. 2011)
hGRE 6.4	AGAACATTTTGTCCG	15	(Alex Yick-Lun So et al. 2007)
hGRE 12.1	AGAACAGAATGTCCT	15	(Alex Yick-Lun So et al. 2007)
hGRE 16.1#1	AGGACAGCCTGTCCT	15	(Alex Yick-Lun So et al. 2007)
hGRE 16.1#2	AGAACAGGATGTTTA	15	(Alex Yick-Lun So et al. 2007)
hGRE 19.2	GGAACACGGCGTCCC	15	(Alex Yick-Lun So et al. 2007)
hGRE 5.1	GGTACATTCTGTTCA	15	(Alex Yick-Lun So et al. 2007)
hGRE 6.1#1	AGAACAGGGTGTTCT	15	(Alex Yick-Lun So et al. 2007)
hGRE 6.1#2	AGCACATCGAGTTCA	15	(Alex Yick-Lun So et al. 2007)
hGRE 6.2	GGTACAGTTTGTTAC	15	(Alex Yick-Lun So et al. 2007)
hGRE 10.3	GGAACAGAAAGTATT	15	(Alex Yick-Lun So et al. 2007)
hGRE 10.4	GGTACAGACCGTTCT	15	(Alex Yick-Lun So et al. 2007)
hGRE 10.5	AGAACACAATGTTCT	15	(Alex Yick-Lun So et al. 2007)
hGRE X.1	AGCACACCCGGAGCA	15	(Alex Yick-Lun So et al. 2007)
hGRE X.2	AGAACATTGGGTTCC	15	(Alex Yick-Lun So et al. 2007)
CYP2C9	aacTGAACTgaaTGTTTTgct	21	(Ferguson SS et al. 2005)
CYP2C9	cccaacTGAACTgaaTGTTTgcttga	26	(Yuping Chen et al. 2003)
CYP2C8	ggtTTAACTggaTGTTTTgct	21	(Ferguson SS et al. 2005)
CYP2C19	ctcaatTGAACTgaaTGTTTTgcttga	27	(Yuping Chen et al. 2003)
GM550	GATCTCTTATTATGACTCTTGCTTTCCTCCTTTCC	35	(Smith PJ et al. 2001)

Supplementary Table S1 (cont.)

A1AR	CAGAACAGTC	10	(Ren H and Stiles GL. 1999)
osteocalcin	agagGGTATAAACAGTGCTggagg	24	(Meyer T et al. 1997)
hElastinGRE1	GTGGCTTCCATGCTGTTCCCTGA	23	(Monaco MD et al. 1997)
hElastinGRE2	CACGACCCTCCCCTGTTCCATCC	23	(Monaco MD et al. 1997)
hElastinGRE3	CTCCCACCTCCCTGTTCCCCCAC	23	(Monaco MD et al. 1997)
hElastinGRE4	TATCAGGAACAGAGTGTTCTGGCG	24	(Monaco MD et al. 1997)
pGRE	AGAACATGATGTTCT	15	(Lundback T and Hard T. 1996)
pGR2	AGGACATGATGTCCT	15	(Lundback T and Hard T. 1996)
pHPV	GCTACATCCTGTTTT	15	Mittal R et al. 1993)
pHPV	GGCACAAAATGTGTT	15	Mittal R et al. 1993)
pHPV	TGTACATTGTGTCAT	15	Mittal R et al. 1993)
HPV Palindrome	AGAACANNNTGTTCT	15	Chan WK et al. 1989)
HPV Consensus	GGTACANNNTGTTCT	15	Chan WK et al. 1989)
HIV	GAGAACA	7	(Ghosh D. 1992)
hGH	GCGCACAATGTGTCCT	16	(Slater EP et al. 1985)
hMTIIA	CGGTACACTGTGTCCT	16	(Slater EP et al. 1985)
MMTV	GGTTACAAACTGTTCT	16	(Slater EP et al. 1985)

Supplementary Table S2

```
MOTIF 1 RGWACABNNTGTYCH

letter-probability matrix: alength= 4 w= 15 nsites= 30 E= 0e+0
0.600000 0.033333 0.300000 0.066667
0.033333 0.033333 0.900000 0.033333
0.633333 0.100000 0.066667 0.200000
1.000000 0.000000 0.000000 0.000000
0.000000 1.000000 0.000000 0.000000
0.966667 0.033333 0.000000 0.000000
0.116667 0.183333 0.316667 0.383333
0.316667 0.183333 0.216667 0.283333
0.383333 0.183333 0.283333 0.150000
0.066667 0.066667 0.100000 0.766667
0.000000 0.000000 1.000000 0.000000
0.033333 0.000000 0.000000 0.966667
0.100000 0.266667 0.066667 0.566667
0.066667 0.800000 0.000000 0.133333
0.166667 0.166667 0.100000 0.566667
```

Supplementary Table S3

Gene	Putative Sites	Size (bp)	Start Position	End position	Strand	Detected by
Clusterin	AGCACAGGAAGTATT	15	132	146	-	FIMO
Clusterin	AACACAATTCTGTTTC	16	51	66	+	In-house script
Clusterin	TGTTTC	6	109	115	+	In-house script
Clusterin	AGATTGTGTCTT	12	276	287	+	In-house script
Clusterin	ACAGGGTGCCGC	12	638	650	+	In-house script
LEDGF/p75	GAAACCCTACGTCCC	15	611	625	-	FIMO
LEDGF/p75	TGTTTT	6	28	33	+	In-house script
LEDGF/p75	AGAACATGAATCTTT	15	315	329	+	In-house script
